# Bayesian estimates of genetic parameters of non-return rate and success in first insemination in Japanese Black cattle

**DOI:** 10.5713/ajas.20.0150

**Published:** 2020-08-30

**Authors:** Asep Setiaji, Daichi Arakaki, Takuro Oikawa

**Affiliations:** 1 Faculty of Animal and Agricultural Sciences, Universitas Diponegoro, Tembalang Campus, Semarang, 50275 Central Java, Indonesia; 2 United Graduate School of Agricultural Sciences, Kagoshima University, 1-21-24 Korimoto, Kagoshima 890-0065, Japan; 3 Faculty of Agriculture, University of the Ryukyus, Nishihara, Okinawa 903-0213 Japan

**Keywords:** Non-return Rate, Success in First Insemination, Heritability, Bayesian Procedure, Japanese Black Cattle

## Abstract

**Objective:**

The objective of present study was to estimate heritability of non-return rate (NRR) and success of first insemination (SFI) by using the Bayesian approach with Gibbs sampling.

**Methods:**

Heifer Traits were denoted as NRR-h and SFI-h, and cow traits as NRR-c and SFI-c. The variance covariance components were estimated using threshold model under Bayesian procedures THRGIBBS1F90.

**Results:**

The SFI was more relevant to evaluating success of insemination because a high percentage of animals that demonstrated no return did not successfully conceive in NRR. Estimated heritability of NRR and SFI in heifers were 0.032 and 0.039 and the corresponding estimates for cows were 0.020 and 0.027. The model showed low values of Geweke (p-value ranging between 0.012 and 0.018) and a low Monte Carlo chain error, indicating that the amount of a posteriori for the heritability estimate was valid for binary traits. Genetic correlation between the same traits among heifers and cows by using the two-trait threshold model were low, 0.485 and 0.591 for NRR and SFI, respectively. High genetic correlations were observed between NRR-h and SFI-h (0.922) and between NRR-c and SFI-c (0.954).

**Conclusion:**

SFI showed slightly higher heritability than NRR but the two traits are genetically correlated. Based on this result, both two could be used for early indicator for evaluate the capacity of cows to conceive.

## INTRODUCTION

The reproductive performance of female Japanese Black cattle has shown a downward trend during the last two decades [1–3]. Selection programs that have focused on carcass traits are thought to be the prime factor for this decline [4]. Thus, the incorporation of reproductive performance in breeding programs is a prerequisite for improving performance traits while maintaining a sustainable beef production system.

In terms of dairy practices in Japan, artificial insemination (AI) is the principal mating method [5]. Success of insemination can be evaluated early by the non-return rate (NRR) within 56 days after the first insemination or the success rate of the first insemination (SFI). Both NRR and SFI have for long been used for evaluating reproductive performance in dairy cattle [6–9]. Nevertheless, only limited studies have been conducted on these two traits for evaluating reproductive performance in Japanese Black cattle.

The NRR could be used to assess the ability of female cattle to conceive and maintain pregnancy during the early period of gestation. The advantage of NRR lies in that it can be measured early and its data are less biased due to fewer missing records than for other reproductive traits [10,11]. The drawback of NRR lies in that cows showing no return within 56 days might or might not be pregnant. Alternatively, SFI can be used as the trait for evaluating the ability of female cattle to conceive and be pregnant. Based on number of records NRR would more heritable than SFI. NRR and SFI should have high genetic correlation because both two traits were binary and measured in the same period.

Theoretically, the threshold model is more relevant than the linear model to estimating genetic parameters of binary trait. The linear model is based on the assumption of normal distribution, whereas the threshold model is based on the assumption of an underlying unobservable continuous response variable that follows the assumptions of normal distribution [12]. On the other hand, the threshold model involves a bias in estimating a variance component when the number of fixed effects is high [13,14]. The objective of this study was to estimate heritability of NRR and SFI by using the Bayesian approach with Gibbs sampling.

## MATERIALS AND METHODS

### Data set

Field records of AI and calving events of heifers and the first three parities of cows were collected from 184 farms. The data set was edited based on the following criteria: cows born between 2004 and 2014, year of insemination between 2005 and 2015 and age, more than 12 months, at first insemination. Eight AI technicians carried out the procedure on all cows. Total animals in the pedigree were 15,600.

The traits studied were NRR and SFI. The NRR was coded 1 when no AI procedure was carried out within 56 days of the first insemination, otherwise 0. SFI was coded 1 if the cow was inseminated only once and subsequently dropped a calf, otherwise 0. Traits of heifers were denoted as NRR-h and SFI-h, and those of cows as NRR-c and SFI-c. Cow with in complete records, embryo transfer donor or recipient, moved from one farm to the other, have twin calves were eliminated to avoid errors of the reproductive recording scheme. The final data set after editing totaled 2,161 heifers and 5,780 cows. The detailed information of the data sets is shown in Table 1.

### Statistical model

The general linear model procedure of Statistical Analysis System 9.3 software [15] was used to test the significant factors of NRR and SFI. The factors tested included farm, year and season of insemination, parity and AI technicians.

The model used for heifer and cow data was

(1) y=Xb+Za+e

And

(2) y=Xb+Za+Wpe+e,

where ***y*** is the vector of observations of NRR of SFI for heifers (model 1) and cows (model 2); ***b*** the vector of fixed effects including farm, season and year of insemination of cows, and AI technician; ***a*** the vector of random additive genetic effect; ***pe*** the vector of permanent environmental effects (only for model 2); and ***e*** the vector of random residuals for the ith trait. ***X***, ***Z***, and ***W*** are incidence matrices connecting ***b***, ***a***, and ***pe*** to ***y***.

The threshold animal model assumed an underlying lia bility (***L***) of NRR and SFI (***y***), the response of observation was modeled after the following distribution:

$$\begin{array}{l} f(y|L)=\prod_{i=1}^{n}f(y_{i}|L_{i})\\ =\prod_{i=1}^{n}\left[I(L_{i}<t_{i})I(y_{i}=0)+I(t_{i}<L_{i}<t_{1})I(y_{i}=1)\right], \end{array}$$

where ***t****_i_* is the threshold that defines the two categories of response and ***I*** the indicator function that takes value 1 if the condition specified is true, otherwise the value is 0. The genetic parameters were estimated through the Bayesian procedure [16] assuming that the normal distribution with density as

$$\begin{array}{l} p(a|\sigma_{a}^{2})\sim N(O,A\sigma_{a}^{2}),\\ p(\mathrm{pe}|\sigma_{pe}^{2})\sim N(O,I\sigma_{pe}^{2})\;\text{and}\\ p(e|\sigma_{e}^{2})\sim N(O,I\sigma_{e}^{2}), \end{array}$$

where ***A*** is the numerator relationship matrix of additive genetic relation between individuals in the pedigree; ***I*** the identity matrix; $\sigma_{a}^{2},\;\sigma_{pe}^{2}$ (only for cows), and $\sigma_{e}^{2}$ the additive genetic variance, permanent environmental variance and residual variance, respectively. The variance covariance components were estimated by using Bayesian procedures THRGIBBS1F90 [17] gave us a period of data collection of 1,000,000 iterations after a burn-in period of 100,000 iterations. Value of burn-in were evaluated through POSTGIBBS1F90 of the Geweke diagnostic test [18]. A posteriori distributios with 4,000 samples were obtained after taken a part for every 250 cycles. Geweke criteria and error of Monte Carlo chain (MCE) used to monitor the convergence, were obtained by calculating the variance of samples for each component divided by the number of samples.

## RESULTS

Farm, year and parity effects were significant in both NRR and SFI, whereas season and AI technician effects were not significant. When data of heifer (first parity) was not included in the analysis, parity effect was not significant (Table 2). Accordingly, subsequent analysis was separated into traits of heifers and cows, and parity effect was excluded from the analysis.

**Figure 1:**
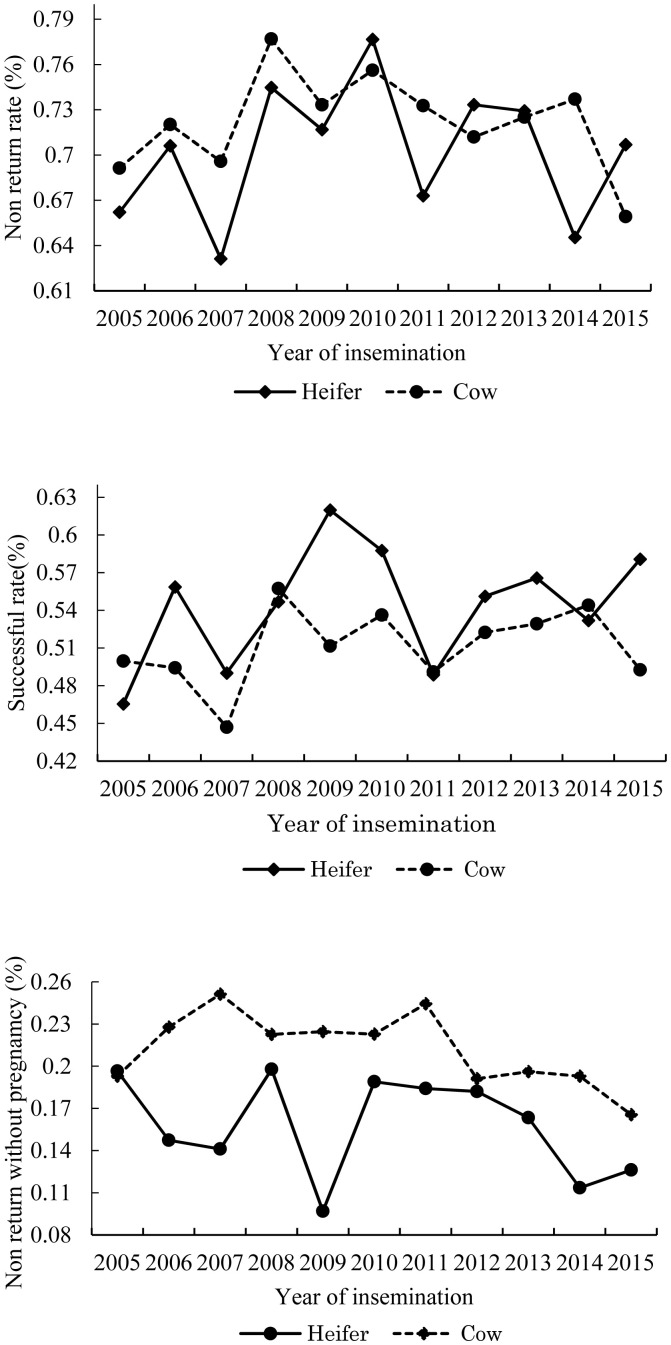
Yearly trend of least square means of non-return rate (NRR) and success at first insemination (SFI).

The highest least square means of the year effect was ob served in 2010 and 2008 for NRR for heifers and cows, respectively. Despite a similar trend shown in heifers and cows, the percentage of NRR for heifers was consistently lower than for cows. The opposite trend was observed in SFI, when heifers tended to show a higher percentage of SFI than did cows (Figure 1).

Estimated heritability of SFI-h was slightly higher than that of NRR-h. The estimated heritability of SFI-c was higher than that of NRR-c (Table 3). In general, estimated heritability by the threshold model showed low posteriori standard deviation. The estimated heritability of all traits in heifers and cows showed a low Geweke value (p-value) ranging between 0.012 and 0.018, MCE ranging between 0.001 and 0.002 also showed a narrow interval of confidence interval of heritability (CI). DIC of estimated heritability ranging between 2,086.90 and 4,683.37 (Table 4).

Genetic correlation between the same traits among heifer and cow by using the two-trait threshold model were low, 0.485 and 0.591 for NRR and SFI, respectively. High genetic correlations were observed between NRR-h and SFI-h (0.922) and between NRR-c and SFI-c (0.954), whereas, genetic correlation between NRR-h with SFI-c and between SFI-h with NRR-c were 0.417 and 0.523, respectively (Table 5).

## DISCUSSION

The yearly trend of NRR in heifers was lower than that in cows, whereas the SFI trend in heifers was higher than that in cows, indicating that heifers have a higher potential for pregnancy at first insemination. The lower percentage of successful first insemination in cows may be caused by physiological factors. Physiological circumstances in female cattle change considerably after the first calving. When cows start producing milk, the metabolic process of steroid hormones increases and thus affects reproductive efficiency, such as low rate of estrus detection [19].

A search of the literature did not provide estimated heri tability of NRR and SFI in beef cattle at present. In the present study, the estimated heritability of NRR and SFI was slightly higher than that reported in dairy cattle, it has ranged between 0.014 and 0.050 and between 0.020 and 0.040 [6,11,20], respectively. The estimated heritability of NRR and SFI in heifers were slightly higher than that in cows (Table 3). This result is interpreted such that the pool of genes affecting the reproductive cycle of an individual and their expression in heifers might be different from that in cows [9].

For Bayesian analysis with the use of Gibbs samplings, low values of the Geweke criterion and MCE for NRR and SFI in both heifers and cows indicated that the amount of posteriori of heritability estimates was valid for binary traits. The low value of MCE indicates that the chain size for particular Bayesian analyses is confirmed as reaching convergence [21]. For the two traits in both heifers and cows, narrow interval of CI and small value of DIC indicating that the model is reliable for estimating NRR and SFI. DIC is a parameter for comparing models, being based on posterior distribution of the likelihood ratio [22].

The low genetic correlation of traits of heifer with traits of cow may be due to several factors. In term of physiological and genetic factors as explained above, it was also effected by farm management. Management, especially feeding have practiced for heifer was different with whose practiced for cow. High genetic corelations between NRR-h and SFI-h and between NRR-c and SFI-c seem to be attributable to the strong correlation between NRR and SFI. Both two traits are binary and indecates the capacity of cows to conceive. This results are consistence with previous studies reported favorable genetic correlations between NRR and traits that recorded before conception (number of inseminations and interval from first to successful insemination) in Japanese Black cows [23] and in Japanese Black heifers [24]. Based on this result, NRR and SFI could be used for early indicator for evaluate the capacity of cows to conceive.

## CONCLUSION

Based on phenotypic trend, SFI was more relevant to evaluating success of insemination because a high percentage of animals that demonstrated no return did not successfully conceive in NRR. Estimated heritability of NRR and SFI was low in both heifers and cows. In general, SFI showed slightly higher heritability than NRR but among two traits are genetically correlated. Based on this result, both two could be used for early indicator for evaluate the capacity of cows to conceive.

## Figures and Tables

**Table 1:** Structure of source data of NRR and SFI

Traits	Heifer	Cow
NRR		
0	742	1,446
1	1,559	4,351
SFI		
0	1,072	2,214
1	1,229	3,583
Total numbers of records	2,301	5,797

NRR, non-return rate; SFI, success at first insemination.

**Table 2:** Significance level of NRR and SFI

Trait	df	NRR		SFI	
	
		F-value	p-value	F-value	p-value
Analysis I^1)^					
Farm	183	1.51	<0.0001	1.82	<0.0001
Year	10	2.95	0.001	2.12	0.0201
Season	3	0.85	0.4645	0.71	0.5448
Parity	3	16.41	<0.0001	17.11	<0.0001
AI technician	7	0.77	0.6846	1.55	0.0975
Analysis II^2)^					
Farm	183	1.71	<0.0001	2.14	<0.0001
Year	10	2.67	0.0034	1.87	0.0449
Season	3	1.59	0.1898	0.80	0.4924
Parity	2	2.21	0.1103	2.83	0.0659
AI technician	7	0.79	0.6617	1.55	0.0983

NRR, non-return rate; SFI, success at first insemination; AI, artificial insemination.

1) All data were included in the analysis.

2) Data of heifers were excluded from the analysis.

**Table 3:** Genetic parameters of NRR and SFI under threshold model

Trait^1)^	$\sigma_{a}^{2}$	$\sigma_{pe}^{2}$	$\sigma_{e}^{2}$	h^2^ (posterior SD)
NRR-h	0.0314	-	0.9421	0.032 (0.038)
SFI-h	0.0375	-	0.9341	0.039 (0.022)
NRR-c	0.0219	0.0329	1.0173	0.020 (0.025)
SFI-c	0.0312	0.0801	1.0249	0.027 (0.013)

$\sigma_{a}^{2}$ , additive genetic variance; $\sigma_{pe}^{2}$, permanent environment variance; $\sigma_{e}^{2}$, residual variance; h^2^, heritability; SD, standard deviation; NRR, non-return rate; SFI, success at first insemination.

1) Heifer traits were denoted as NRR-h and SFI-h, and cow traits as NRR-c and SFI-c.

**Table 4:** Monte Carlo error, confidence interval of heritabilityand deviance information criterion for heritability estimates under threshold model

Trait^1)^	Geweke (p-value)	MCE	CI	DIC
NRR-h	0.015	0.001	0.016–0.045	2,086.90
SFI-h	0.018	0.002	0.018–0.050	2,401.25
NRR-c	0.012	0.002	0.008–0.032	4,618.09
SFI-c	0.014	0.002	0.012–0.031	4,683.37

MCE, Monte Carlo error; CI, confidence interval of heritability; DIC, deviance information criterion of heritability; NRR, non-return rate; SFI, success at first insemination.

1) Heifer Traits were denoted as NRR-h and SFI-h, and cow traits as NRR-c and SFI-c.

**Table 5:** Genetic correlations (above the diagonal) posterior standard deviation (below the diagonal) between NRR and SFI under threshold model

Trait^1)^	NRR-h	SFI-h	NRR-c	SFI-c
NRR-h	-	0.922	0.485	0.417
SFI-h	0.018	-	0.523	0.591
NRR-c	0.021	0.035	-	0.954
SFI-c	0.032	0.025	0.016	-

NRR, non-return rate; SFI, success at first insemination.

1) Heifer Traits were denoted as NRR-h and SFI-h, and cow traits as NRR-c and SFI-c.
